# Spontaneous population activity fluctuations boost sensory tuning curves and gate information processing

**DOI:** 10.1186/1471-2202-14-S1-P281

**Published:** 2013-07-08

**Authors:** Iñigo Arandia-Romero, Jan Drugowitsch, Adam Kohn, Rubén Moreno-Bote

**Affiliations:** 1 Research Unit, Parc Sanitari Sant Joan de Deu and Universitat de Barcelona, Esplugues de Llobregat, Barcelona, 08950, Spain; 2 Centro de Investigación Biomédica en Red de Salud Mental (CIBERSAM), Esplugues de Llobregat, Barcelona, 08950, Spain; 3 Institut National de la Santé et de la Recherche Médicale & École Normale Supérieure, Paris, 75005, France; 4 Dominick Purpura Department of Neuroscience; 5 Ophthalmology and Visual Science, Albert Einstein College of Medicine, Bronx, New York, 10461, USA

## Background

Sensory cortex often undergoes population activity fluctuations due to attentional modulations and arousal changes, but activity fluctuations can also be generated intrinsically. The mechanisms that underlie these spontaneous activity fluctuations are unknown, in particular whether they arise from common inputs or more active neuronal processes. We measured the activity of tens of V1 neurons simultaneously in monkeys stimulated with oriented visual stimuli, and found that the tuning curves (TCs) of orientation selective neurons spontaneously underwent shifts and multiplicative scalings proportional to population activity while their widths remained constant Based on this observed TC activity dependence, we constructed a precise statistical model featuring Poisson-like firing, shifts and gain modulation. The model not only accounted for neuronal co-variability but also approached the performance of state-of-art decoding methods. Surprisingly, we found that decoding performance on sensory stimuli increased with population activity, despite the fact that the stimulus was identical. Therefore, spontaneous population activity fluctuations display highly non-random features, boosting TCs by shifts and multiplicative factors that gate information processing.

## Results

**Figure 1:**
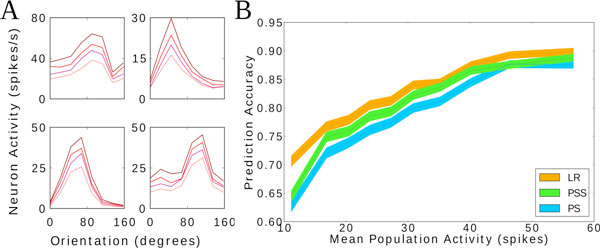
A: TCs conditioned to four different mean population activity bins, ranging from high mean population activity (upper line) to low mean population activity (lower line). B: Decoding performance of stimulus orientation as a function of mean population activity for four different decoding models.

We found that TCs are shifted and scaled by a multiplicative factor proportional to mean population activity (Figure 1A). The amount of each contribution was neuron dependent. We studied whether the scaling in the TCs induced by population activity fluctuations had an impact on information processing. To this end, we decoded orientation on a trial-by-trial basis from neuronal activity as a function of population activity (Figure 1B). We found that higher mean population activity resulted in better decoding accuracy. This result is surprising because the amount of information conveyed by V1 neurons, even for the same stimulus, depends on the mean population activity.

## Methods

The observed boosting properties of TCs motivated us to build a statistical model with Poisson-like neurons that includes a multiplicative scaling factor (PS). We observed that the effect of population activity on TCs is not purely multiplicative, and we introduced therefore a shift (PSS). Our models approached the performance of state-of-art decoding techniques (SVM, and logistic regression -LR-) and provided higher accuracy than other tested decoders based on population vector and independent Poisson neurons. Experimental methods are as in [1].

## References

[B1] Smith MA Kohn A Spatial and Temporal Scales of Neuronal Correlation in Primary Visual Cortex J Neurosci 2008 28 48 12591 603 10.1523/JNEUROSCI.2929-08.2008 19036953 PMC2656500

